# Two-Stage Dynamic Synergistic Segmentation Method for Myocardial Pathology

**DOI:** 10.3390/jimaging12060269

**Published:** 2026-06-18

**Authors:** Dongsheng Ruan, Xiaolin Zhang, Zihan Yuan, Ziqian Lu, Ling Xia, Mingfeng Jiang

**Affiliations:** 1 Department of Computer Science and Technology, Zhejiang Sci-Tech University, Hangzhou 310018, China; ruandongsheng@zstu.edu.cn (D.R.); 2023220603101@mails.zstu.edu.cn (X.Z.); 2023110602006@mails.zstu.edu.cn (Z.Y.); ziqianlu@zstu.edu.cn (Z.L.); 2 Department of Biomedical Engineering, Zhejiang University, Hangzhou 310018, China; xialing@zju.edu.cn

**Keywords:** myocardial pathology segmentation, multi-modal cardiac MRI, small object segmentation, class imbalance, UNet

## Abstract

Myocardial scar and edema segmentation from multi-sequence cardiac magnetic resonance (MS-CMR) is important for myocardial infarction assessment, but remains challenging due to heterogeneous modal characteristics, severe class imbalance, and the small, ambiguous nature of pathological regions. To address these issues, a dynamic synergistic segmentation network (DSS-Net) is proposed for myocardial pathology segmentation. The framework adopts a coarse-to-fine strategy, in which a coarse stage first segments the myocardium to provide anatomical priors and region constraints, and a fine stage then delineates scar and edema within the myocardium-aware space. In addition, a Modality Dynamic Fusion Module (MDFM) is designed to adaptively emphasize pathology-relevant modal information, and a Stage Feature Aggregation Module (SFAM) is introduced to enhance cross-stage feature interactions and fine-grained lesion representation. Experiments on the MyoPS 2020 and MyoPS 2024 datasets demonstrate that DSS-Net achieves competitive and balanced performance, reaching Dice scores of 0.706 for scar and 0.753 for edema on MyoPS 2020. Additionally, compared with SOTA methods in the MyoPS 2020 Challenge, the proposed method attains comparable scar segmentation performance while maintaining a more balanced trade-off between sensitivity and specificity. These findings suggest that combining anatomical guidance with pathology-aware multi-modal learning is a promising strategy for robust myocardial pathology segmentation in MS-CMR images.

## 1. Introduction

Myocardial infarction (MI) remains one of the leading causes of morbidity and mortality worldwide and poses a substantial burden on public health systems [1]. Following ischemic injury, a series of pathological changes may occur within the myocardium, among which myocardial scar and edema are two important imaging manifestations closely associated with tissue viability, infarct severity, and clinical prognosis [2]. Accurate delineation of these pathological regions is therefore of great significance for diagnosis [3], treatment planning, and post-infarction risk stratification. Cardiac magnetic resonance (CMR), especially multi-sequence CMR (MS-CMR), provides complementary information for cardiac tissue characterization and has become an important imaging tool for myocardial pathology assessment [4]. Compared with single-modality imaging, MS-CMR can describe myocardial abnormalities from different perspectives [5]. For example, different sequences may exhibit distinct sensitivities to edema, scar, and normal myocardium, thereby offering complementary cues for lesion identification. This property makes MS-CMR particularly promising for automated segmentation of myocardial pathology.

Recent advances in deep learning have significantly improved medical image segmentation performance [6,7], and a variety of convolutional neural networks and hybrid architectures have been introduced for cardiac image analysis. Existing studies have demonstrated the potential of deep models for segmenting cardiac structures and pathological tissues from CMR images. Nevertheless, accurate segmentation of myocardial scar and edema from MS-CMR remains far from solved. One important reason lies in how to exploit multi-modal information effectively. Although different CMR sequences provide complementary cues for tissue characterization, their contributions to lesion delineation are often unequal and spatially variable. In practice, certain modalities may be more informative for edema, whereas others may better highlight scar or myocardial boundaries [8]. However, many existing methods rely on straightforward input concatenation or static feature fusion strategies [9,10], which may introduce redundant or even conflicting information and cannot adaptively emphasize the modality cues most relevant to the target pathology. As a result, the full diagnostic value of MS-CMR is not always adequately utilized. Another major challenge is that myocardial scar and edema segmentation is intrinsically difficult because these lesions often occupy only small subregions within the myocardium and may exhibit irregular morphology, blurred boundaries, and severe class imbalance relative to the background and healthy tissue [11]. These characteristics make the lesions susceptible to being overlooked during repeated down-sampling and feature transformation, leading to missed detections, fragmented segmentation, and inaccurate boundary delineation. Therefore, enhancing the representation of small and subtle pathological targets remains a key issue for robust myocardial pathology segmentation.

To address these challenges, this study proposes a Dynamic Synergistic Segmentation Network (DSS-Net) for myocardial pathology segmentation from MS-CMR images. The proposed method adopts a unified two-stage segmentation framework. In the first stage, a coarse segmentation network generates myocardium priors by segmenting the myocardial structure and localizing pathology-related myocardial regions. In the second stage, a fine segmentation network takes both the MS-CMR images and the generated myocardium prior as inputs to refine the segmentation of scar and edema. Furthermore, a Modality Dynamic Fusion Module (MDFM) is introduced to adaptively exploit complementary information across different imaging modalities, while a Stage-wise Feature Aggregation Module (SFAM) is designed to strengthen fine-grained lesion representation and improve the delineation of small pathological targets.

The main contributions of this work are summarized as follows:

- We propose a unified two-stage dynamic synergistic segmentation network for myocardial pathology segmentation in MS-CMR images, which integrates myocardium-aware coarse localization and fine lesion delineation within an end-to-end optimization framework.
- We design a modality dynamic fusion module to adaptively select and fuse pathology-relevant information from different CMR modalities, thereby improving the utilization of complementary multi-modal cues.
- We introduce a stage feature aggregation module to enhance the representation of small and subtle lesions, which improves the segmentation of myocardial scar and edema.

## 2. Related Work

### 2.1. Deep Learning-Based Segmentation Methods

Image segmentation is a fundamental task in medical image analysis. Traditional methods, such as thresholding [12], region growing [13], graph-cuts [14], and level-set-based algorithms [15], can achieve satisfactory performance in relatively simple settings, but their reliance on handcrafted features and manually tuned prior assumptions limits their robustness in complex clinical scenarios. With the development of deep learning, image segmentation has gradually shifted from rule-based modeling to data-driven feature learning.

The Fully Convolutional Network (FCN) [16] established an end-to-end dense prediction paradigm by replacing fully connected layers with convolutional operations, making pixel-wise semantic segmentation feasible within a unified framework. SegNet [17] adopted an encoder-decoder architecture and reused pooling indices during decoding, which improved the recovery of spatial structures and object boundaries. U-Net [18] further enhanced medical image segmentation by introducing skip connections between encoder and decoder stages, enabling the fusion of low-level spatial details and high-level semantic features. DeepLab [19] expanded the receptive field through atrous convolution and improved multi-scale context modeling, while its CRF-based refinement strengthened boundary delineation. To reduce the semantic gap between encoder and decoder features, UNet++ [20] redesigned skip pathways with nested dense connections, leading to improved feature aggregation. Attention U-Net [21] incorporated attention gates to suppress irrelevant responses and emphasize target-related regions, thereby improving structural localization. More recently, Transformer-based models such as TransUNet [22] and Swin-Unet [23] have introduced global self-attention or hierarchical Transformer encoding into the U-shaped framework, enhancing long-range dependency modeling and contextual representation. Although these methods have substantially advanced general medical image segmentation, they are mainly designed for common anatomical structures and often remain inadequate for subtle pathological targets with irregular shapes, ambiguous boundaries, and limited spatial extent.

### 2.2. Multi-Modal Segmentation Methods

Multi-modal segmentation aims to improve delineation performance by exploiting the complementary information provided by different imaging modalities, since a single modality often captures only part of the anatomical or pathological characteristics. Existing multi-modal fusion methods can generally be divided into early fusion, intermediate fusion, and decision fusion, depending on the stage at which information from different modalities is integrated.

Early fusion strategies [9,10,24] directly concatenate images from different modalities as multi-channel inputs, requiring only minimal architectural modification and treating all modalities equally. For example, DPAFNet [25] concatenates multi-modal inputs and enhances their integration through dual-path learning and multi-scale attention fusion, thereby improving segmentation accuracy. Intermediate fusion strategies [26,27] encode each modality separately and then merge them in the latent space, which preserves modality-specific features before fusion and focuses more on architectural design. For instance, Xu et al. [28] proposed a knowledge-guided multi-modal fusion method that employs a multi-phase self-attention module to fully exploit cross-modal information. Decision fusion methods [29,30] process different modalities through independent branches or networks and combine their predictions at the output stage. Guo et al. [31] obtained the final segmentation result by averaging the outputs of modality-specific convolutional networks, thereby improving segmentation accuracy. Although these fusion strategies have demonstrated the effectiveness of multi-modal information for medical image segmentation, most of them rely on predefined or empirically designed fusion mechanisms. Consequently, they often fail to adaptively emphasize the most informative modality cues when modality contributions vary across spatial locations and target characteristics.

### 2.3. Feature Fusion Methods

Feature fusion has been widely studied in image segmentation. Existing fusion methods mainly focus on combining features from different network levels or different representation spaces. Representative feature fusion methods include VeinMask [32], where hierarchical features are progressively integrated to improve object representation. FEPE [33] enhances feature interaction through the joint modeling of instance-level coherence and contextual information. To facilitate multi-scale feature learning, ZTD [34] combines global localization with local refinement via a zooming mechanism. CM-Net [35] further improves representation capability by aggregating complementary information from multiple feature levels. Although these methods have demonstrated the effectiveness of feature fusion, they are primarily designed to improve feature interactions among different network layers. In contrast, myocardial pathology segmentation from multi-sequence CMR images requires effective integration of complementary information from different imaging modalities. Since different modalities provide distinct pathological characteristics, simply aggregating features from multiple sources may introduce redundant or even conflicting information. To this end, we propose MDFM and SFAM, which are designed to support adaptive cross-modal fusion and semantic-aware feature aggregation in multi-sequence CMR segmentation.

### 2.4. Myocardial Pathology Segmentation Methods

Myocardial pathological regions are usually confined to limited areas within the myocardium and often exhibit severe class imbalance, which makes accurate delineation particularly difficult. Existing studies on small-target segmentation mainly explore three directions: loss function design [36,37,38], multi-scale feature learning [39,40,41], and multi-stage segmentation [11,42,43]. For instance, specialized loss functions such as Dice loss have been introduced to alleviate class imbalance. Multi-scale convolution integrates local details and global contextual information to enhance the representation of subtle pathological structures. Multi-stage strategies adopt a coarse-to-fine manner, in which the target region is first roughly localized and the segmentation result is then progressively refined.

These ideas have also influenced the development of myocardial pathology segmentation methods. More recently, with the emergence of multi-sequence CMR datasets and the increasing modeling capability of deep learning, myocardial pathology segmentation has evolved from single-pathology, single-modality analysis to joint segmentation of scar and edema from multi-sequence CMR images. To better exploit complementary pathology-related information across sequences, a series of methods have explored multi-sequence feature fusion, including shared-encoder frameworks [44], multi-layer fusion strategies [45], and anatomically guided two-stage pipelines [46]. In particular, coarse-to-fine strategies have become increasingly common in the MyoPS task [44,47,48], where the first stage segments the cardiac anatomy or myocardium and the second stage focuses on pathological regions within the anatomically constrained area. Such designs improve lesion localization and reduce interference from irrelevant background structures. Inspired by these advances, our method adopts a multi-stage, pathology-target-enhanced framework to alleviate the severe class imbalance issue in myocardial pathology segmentation.

## 3. Materials and Methods

### 3.1. Coarse Segmentation Network

Since both scar and edema regions are confined within the myocardium, directly performing segmentation over the entire image introduces substantial background redundancy and exacerbates the class imbalance induced by pathological tissues. To address this issue, the proposed DSS-Net incorporates a coarse segmentation stage, termed Dynamic Synergistic Segmentation Coarse Stage (DSS-Coarse), to localize the myocardium and the left ventricle (LV). This stage provides essential anatomical priors for subsequent pathological delineation, thereby enhancing the network’s focus on clinically relevant regions and improving the representation of pathological structures.

Given that this stage primarily focuses on anatomical localization, a standard U-Net is adopted as the coarse segmentation backbone. Owing to its structural simplicity, stable optimization behavior, and strong empirical performance in medical image segmentation, U-Net provides an efficient and reliable solution. Specifically, its skip connections effectively fuse low-level spatial details with high-level semantic representations, enabling accurate localization while avoiding unnecessary architectural complexity.

**Figure 1:**
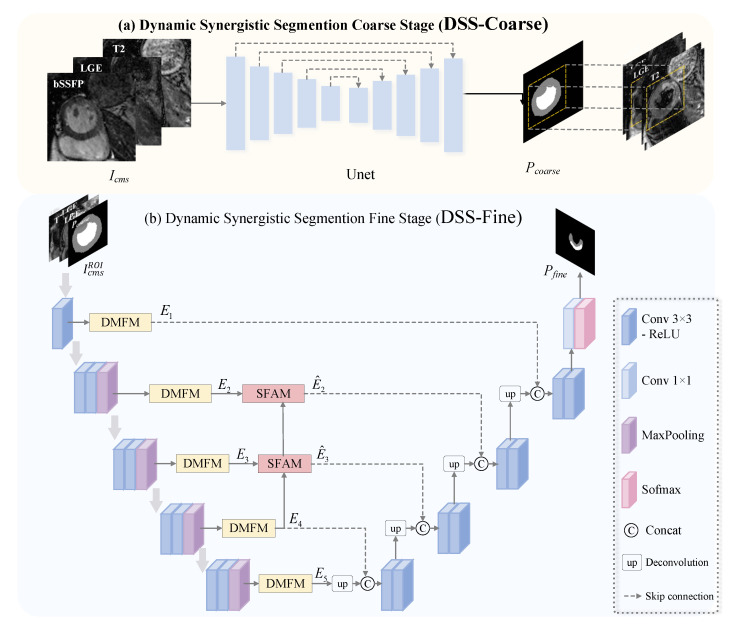
Our proposed dynamic synergistic segmentation network (DSS-Net). DSS-Coarse takes *bSSFP*, *LGE*, and *T*2 images as inputs, outputs the corresponding cardiac structure segmentation map $P_{coarse}$, and determines the myocardial ROI. In the fine segmentation stage, DSS-Fine further segments myocardial scar and edema from multi-sequence CMR within the ROI. MDFM denotes the Modality Dynamic Fusion Module, and SFAM denotes the Stage-wise Feature Aggregation Module. The features enhanced by MDFM are denoted as $E_{i}$, and the features enhanced by SFAM are denoted as ${\hat{E}}_{i}$. Both $E_{i}$ and ${\hat{E}}_{i}$ are connected to the decoder through skip connections.

As illustrated in Figure 1a, the *bSSFP*, *LGE*, and *T*2 cardiac magnetic resonance images for each patient are represented as $I_{mod}\in R^{C\times H\times W},mod\in \left\{bSSFP,LGE,T2\right\}$, where *C* denotes the number of feature channels and H×W represents the spatial dimensions. The input $I_{mod}$ is then fed into the U-Net to predict the probability maps of the myocardium and the left ventricle.

(1) $$P_{coarse}=P_{myo},P_{lv}=\mathrm{DSS-Coarse}\left(I_{bSSFP},I_{LGE},I_{T2}\right).$$

To reduce the region of interest (ROI) and alleviate class imbalance, a myocardium-centered ROI is constructed. Specifically, the predicted probability map is binarized to obtain a foreground mask, from which a bounding box is extracted and further expanded with a fixed margin to preserve anatomical integrity. The resulting ROI is then used to consistently crop all input modalities, serving as a preprocessing step before fine-grained pathological segmentation.

### 3.2. Myocardial Infarction Fine Segmentation Network

To refine the coarse segmentation results, a fine segmentation stage, termed Dynamic Synergistic Segmentation Fine Stage (DSS-Fine), is further introduced. Within the ROI region generated by the DSS-Coarse stage, a dedicated fine-grained segmentation network is employed to predict scar and edema with enhanced precision.

The DSS-Fine stage is built upon a U-Net architecture, as illustrated in Figure 1b. The encoder consists of four parallel branches corresponding to *LGE*, *T*2, and two prior inputs, which are denoted as $I_{mod}^{\mathrm{ROI}}\in R^{C\times h\times w}mod\in \left\{LGE,T2,P_{myo},P_{lv}\right\}$, where *C* denotes the number of feature channels and h×w represents the spatial dimensions after cropping. At each stage, modality-specific features are first extracted independently to preserve the heterogeneous imaging characteristics across different modalities. To better exploit complementary pathological cues across modalities, a Modality Dynamic Fusion Module (MDFM) is introduced to perform adaptive multi-modal feature fusion at each stage. Furthermore, considering that intermediate features retain relatively rich spatial details, a Stage Feature Aggregation Module (SFAM) is incorporated to enhance both local and global representations of small pathological regions. Detailed implementations of MDFM and SFAM are provided in Section 3.3 and Section 3.4, respectively.

In the first stage, $I_{mod}^{\mathrm{ROI}}$ are first fed into four modality-specific backbone blocks to extract shallow features while preserving the distinct appearance characteristics of each branch. Subsequently, these features are fused via the MDFM to generate the first-stage encoded representation:

(2) $$X_{mod}^{1}=\delta \left(Conv\left(I_{mod}^{\mathrm{ROI}}\right)\right),$$

(3) $$E_{1}=\mathrm{MDFM}\left(X_{mod}^{1}\right)$$

where $X_{mod}^{1}$ denotes the modality-specific encoded features, $mod\in \left\{LGE,T2,P_{myo},P_{lv}\right\}$, and $E_{1}$ represents the encoded features after multi-modal fusion. $Conv\left(⬝\right)$ denotes a 2D convolution with a kernel size of 3, and $\delta \left(⬝\right)$ refers to the ReLU activation function.

From the second stage onward, each branch follows the same encoding procedure. The feature extraction at stage $i\in \left\{2,3,4,5\right\}$ can be formulated as:

(4) $$X_{mod}^{i}=F\left(X_{mod}^{i-1}\right)$$

(5) $$E_{i}=\mathrm{MDFM}\left(X_{mod}^{i}\right)$$

where $F\left(⬝\right)$ denotes the feature encoding operation, which consists of convolution, nonlinear activation, and max-pooling, progressively enlarging the receptive field. Subsequently, the MDFM adaptively fuses the four modality-specific features to produce $E_{i}$. This dynamic fusion mechanism enables the network to emphasize modality-specific cues that are more discriminative for scar and edema at different semantic levels, while suppressing redundant responses, thereby improving the representation of ambiguous pathological regions.

To further enhance the representation of small targets, the SFAM is introduced after modality fusion at intermediate stages, where high-level semantic cues are injected into relatively high-resolution intermediate features. This process can be formulated as:

(6) $${\hat{E}}_{i}=\mathrm{SFAM}\left(E_{i},E_{i+1}\right),\;i=3,2$$

Subsequently, the decoder takes the encoded features $E_{i}/{\hat{E}}_{i}$ as input and progressively restores the spatial resolution starting from the deepest feature representation $E_{m}^{5}$ through a series of upsampling and skip-connection fusion operations. Based on the final decoder feature $d_{2}$, the predictions for scar and edema are generated as:

(7) $$d_{i}=D_{i}\left(\left[Up\left(E_{i}\right)E_{i-1}\right]\right),i=5,4,3,2$$

(8) $$P_{scar}=\sigma \left(Conv_{1\times 1}\left(d_{2}\right)\right)$$

(9) $$P_{edema}=\sigma \left(Conv_{1\times 1}\left(d_{2}\right)\right)$$

where $Up\left(\cdot \right)$ denotes the upsampling convolution operation, $D_{i}\left(\cdot \right)$ represents the convolutional decoding block at stage *i*, $Conv_{1\times 1}\left(\cdot \right)$ denotes a convolutional layer with a kernel size of 1, and $\sigma \left(\cdot \right)$ refers to the Softmax activation function.

### 3.3. Modality Dynamic Fusion Module

Different cardiac magnetic resonance (CMR) modalities contribute unequally to myocardial pathology segmentation. Specifically, *LGE* is more sensitive to scar regions, whereas *T*2 better highlights abnormalities associated with edema. Direct feature fusion neglects such modality-specific preferences and may attenuate responses relevant to pathological tissues.

To address this limitation, we propose a Modality Dynamic Fusion Module (MDFM), which explicitly models modality-specific features prior to fusion. By learning inter-modality correlations, the module enables the network to selectively enhance informative features from each modality, thereby better exploiting their complementary characteristics. Furthermore, to allow the fusion process to emphasize modality-specific responses at different spatial locations, MDFM is implemented in a fully convolutional manner.

**Figure 2:**
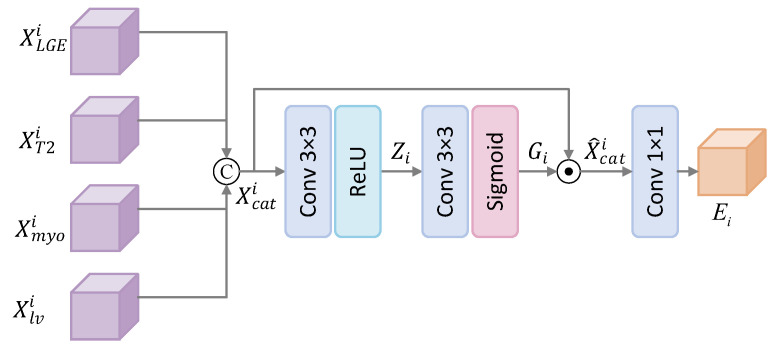
Structure of the proposed Modality Dynamic Fusion Module (MDFM).

The architecture of MDFM is illustrated in Figure 2. At stage *i*, the input to MDFM is given by $X_{mod}^{i}\in R^{C^{i}\times h^{i}\times w^{i}}$, where $C^{i}$ denotes the number of feature channels and $h^{i}\times w^{i}$ represents the spatial dimensions, corresponding to *LGE*, *T*2, and two anatomical prior features. These modality-specific features $X_{mod}^{i}$ are first concatenated to form a joint representation $X_{cat}^{i}\in R^{4C^{i}\times h^{i}\times w^{i}}$. Prior to fusion, $X_{cat}^{i}$ is recalibrated via a bottleneck gating branch. This branch consists of two convolutional layers. The first layer performs channel reduction to compress the joint representation and enhance its capacity for correlation modeling, while the second layer projects the compressed features back to the original concatenated channel dimension and applies a Sigmoid activation to generate a modulation map. The gating generation process can be formulated as:

(10) $$Z_{i}=\delta \left(W_{1}^{i}*X_{cat}^{i}\right)$$

(11) $$G_{i}=\sigma \left(W_{2}^{i}*Z_{i}\right)$$

where $W_{1}^{i}\in R^{\frac{C^{i}}{\eta }\times h^{i}\times w^{i}}$ denotes the weights of the first convolutional layer, η represents the channel reduction ratio, and $\delta \left(⬝\right)$ refers to the ReLU activation function that introduces nonlinearity. $W_{2}^{i}\in R^{4C^{i}\times h^{i}\times w^{i}}$ denotes the weights of the second convolutional layer, and $\sigma \left(\cdot \right)$ represents the Sigmoid function, which scales the activation values to the range [0, 1].

The generated modulation map is then used to recalibrate $X_{cat}^{i}$, whereby modality-specific features that are more relevant to scar or edema at the current stage are selectively enhanced, while less informative or redundant responses are suppressed. Finally, the recalibrated features are fused through a convolutional projection layer to produce the output representation:

(12) $${\hat{X}}_{cat}^{i}=X_{cat}^{i}⊙G_{i}$$

(13) $$E_{i}=W_{3}^{i}*{\hat{X}}_{cat}^{i}$$

where ⊙ denotes element-wise multiplication, $W_{3}^{i}\in R^{C^{i}\times h^{i}\times w^{i}}$ represents the weights of the third 1×1 convolutional layer followed by batch normalization, and the final output $E_{i}$ is utilized as the skip connection feature for the decoder.

### 3.4. Stage-Wise Feature Aggregation Module

During the hierarchical encoding process, small pathological regions are particularly susceptible to information attenuation. Although high-level features provide stronger semantic representations, their reduced spatial resolution may compromise the delineation of fine-grained lesion structures. In contrast, intermediate features preserve richer spatial details. To better balance local detail and semantic context, we introduce a Stage Feature Aggregation Module (SFAM), which adaptively aggregates features from adjacent stages to enhance the representation at the current stage. Furthermore, prior studies have shown that large-kernel convolutions can suppress redundant image responses while providing a broader receptive field. Compared with repeatedly stacking small-kernel convolutions, large-kernel operations capture wider contextual information more directly, thereby alleviating the loss of pathological details during encoding. Accordingly, SFAM incorporates an additional large-receptive-field branch to strengthen lesion-related responses and improve the segmentation of ambiguous pathological regions.

**Figure 3:**
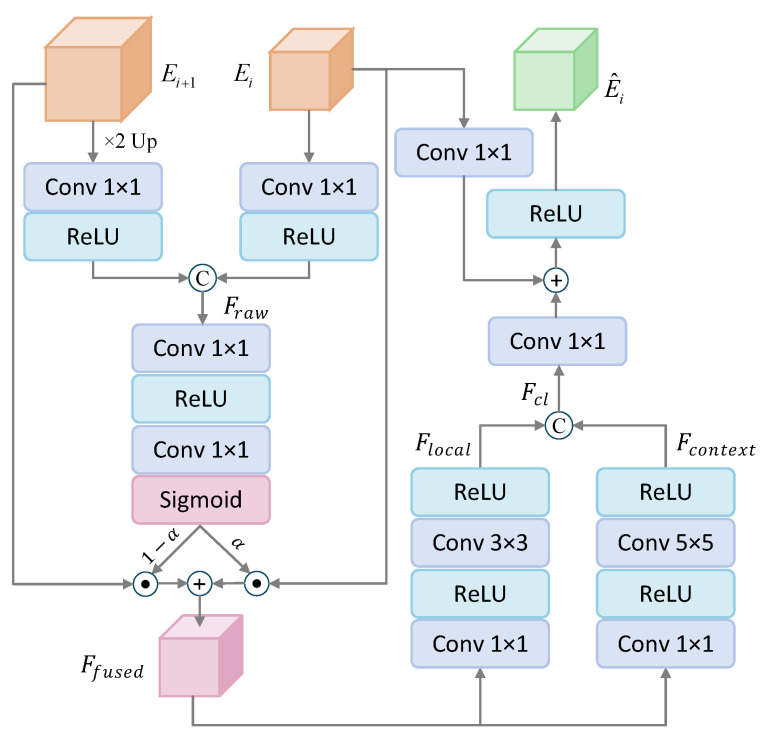
Structure of the proposed Stage-wise Feature Aggregation Module (SFAM).

As illustrated in Figure 3, given the feature $E_{i}\in R^{C^{i}\times h^{i}\times w^{i}}$ at stage *i*, and the adjacent higher-level feature $E_{i+1}\in R^{C^{i+1}\times h^{i+1}\times w^{i+1}}$, they are first aligned to the same dimensional space via convolution and interpolation operations, and then concatenated to form an initial representation $F_{raw}$. Subsequently, a cross-stage selection mechanism is employed to adaptively select informative features for fusion, which can be formulated as:

(14) $$\alpha =\sigma (\psi \left(F_{raw}\right))$$

(15) $$F_{fused}=\alpha ⊙E_{i}+\left(1-\alpha \right)⊙E_{i+1}$$

where $\psi \left(⬝\right)$ denotes two consecutive 1×1 convolutional layers followed by nonlinear ReLU activations, $\sigma \left(⬝\right)$ represents the Sigmoid activation function, and ⊙ denotes element-wise multiplication. Through $\alpha \in R^{C^{i}\times h^{i}\times w^{i}}$, $F_{fused}$ is adaptively modulated to achieve an effective fusion of fine-grained pathological details and high-level semantic information.

For $F_{fused}$, two parallel branches are employed for feature processing. A local branch is designed to capture fine-grained spatial structures, while the other branch utilizes large-kernel convolutions to model complex global context within an expanded receptive field.

(16) $$F_{local}=Conv_{3\times 3}\left(Conv_{1\times 1}\left(F_{fused}\right)\right)$$

(17) $$F_{context}=Conv_{5\times 5}\left(Conv_{1\times 1}\left(F_{fused}\right)\right)$$

(18) $$F_{cl}=concat\left(F_{local},F_{context}\right)$$

where $Conv_{3\times 3}$ and $Conv_{5\times 5}$ denote convolutional operations with kernel sizes of 3 and 5, respectively, each followed by a ReLU activation. The resulting features with different receptive fields are then concatenated along the channel dimension to produce a hybrid representation that integrates multi-scale contextual information.

Finally, the enhanced feature $F_{cl}$, regarded as a pathology-enhanced representation, is combined with the initial stage feature $E_{i}$ via a residual connection. This process can be formulated as:

(19) $${\hat{E}}_{i}=\delta \left(B\left(Conv_{1\times 1}\left(E_{m}^{i}\right)\right)+B\left(Conv_{1\times 1}\left(F_{cl}\right)\right)\right)$$

where $Conv_{3\times 3}\left(⬝\right)$, $B\left(⬝\right)$, and $\delta \left(⬝\right)$ denote convolution, batch normalization, and the ReLU activation function, respectively. The final output of the SFAM module is denoted as ${\hat{E}}_{i}\in R^{C^{i}\times h^{i}\times w^{i}}$. At encoding stages where SFAM is applied, ${\hat{E}}_{i}$ is propagated to the decoder via skip connections, whereas at the remaining stages, the features $E_{i}$ are directly forwarded without additional aggregation.

### 3.5. Loss Function

DSS-Net is trained in an end-to-end manner, jointly optimizing both coarse anatomical segmentation and fine-grained pathological segmentation. The overall loss function is defined as:

(20) $$L_{total}=\lambda_{s}L_{seg}+\lambda_{se}L_{se}$$

where $L_{seg}$ denote the loss terms for the DSS-Coarse and DSS-Fine segmentation results, respectively, $L_{se}$ represents the inclusion constraint enforcing that scar regions are contained within edema, and $\lambda_{s}$ and $\lambda_{se}$ are balancing coefficients.

For the segmentation loss, a composite loss function Dice is adopted by combining Dice loss and Cross-Entropy (CE) loss, which is applied to the segmentation of the myocardium and left ventricle, as well as scar and edema. It is defined as:

(21) $$L_{seg}=\mathrm{Dice}\left(S_{cardiac},L_{cardiac}\right)+2\mathrm{Dice}\left(S_{scar},L_{scar}\right)+2\mathrm{Dice}\left(S_{edema},L_{edema}\right)$$

where $S_{ana}$ denotes the predicted segmentation map and $L_{ana}$ represents the corresponding ground-truth labels, $ana\in \left\{cardiac,scar,edema\right\}$. Considering that scar and edema regions typically occupy relatively small volumes and are considerably more challenging to segment than myocardial structures, higher weights are assigned to these classes in the loss function (with a weighting factor of 2). This design encourages the model to focus more on small pathological regions, thereby improving its discriminative capability and robustness in fine-grained pathology segmentation tasks.

Building upon the segmentation loss, we further incorporate prior knowledge of myocardial pathological structures by introducing a scar–edema inclusion constraint loss, denoted as $L_{se}$. From a clinical perspective, these two pathological regions exhibit a clear spatial inclusion relationship, where scar regions are typically located within or highly overlapped with edema regions. Accordingly, $L_{se}$ is designed to enforce the relative spatial consistency between scar and edema, thereby reducing anatomically implausible predictions and improving the structural coherence of the segmentation results. Specifically, $L_{se}$ consists of two complementary components: $L_{scar}^{inc}$ suppresses scar responses in regions that are not labeled as edema, constraining scar predictions from extending beyond the edema region; conversely, $L_{edema}^{inc}$ enhances edema responses within the ground-truth scar regions, ensuring that scar areas are effectively covered by edema. The formulation is given as:

(22) $$L_{se}=L_{scar}^{inc}+L_{edema}^{inc}$$

(23) $$L_{scar}^{inc}=-\frac{\sum_{i}\left(1-L_{edema}^{\left(i\right)}\right)\log\left(1-S_{scar}^{\left(i\right)}+\epsilon \right)}{\sum_{i}\left(1-L_{edema}^{\left(i\right)}\right)+\epsilon }$$

(24) $$L_{edema}^{inc}=-\frac{\sum_{i}L_{scar}^{\left(i\right)}\log\left(S_{edema}^{\left(i\right)}+\epsilon \right)}{\sum_{i}L_{scar}^{\left(i\right)}+\epsilon }$$

where $S_{scar}^{\left(i\right)}$ and $S_{edema}^{\left(i\right)}$ denote the predicted probabilities of scar and edema at the *i* pixel, respectively, while $L_{scar}^{\left(i\right)}$ and $L_{edema}^{\left(i\right)}$ represent the corresponding ground-truth labels. A small constant ϵ is introduced to avoid numerical instability in logarithmic operations and division. The combination of $L_{scar}^{inc}$ and $L_{edema}^{inc}$ encourages the model to learn pathology distributions that conform to established medical priors, thereby improving the structural plausibility and clinical interpretability of the segmentation results.

## 4. Experiment

### 4.1. Datasets and Preprocessing

We conducted experiments on two public datasets, MyoPS 2020 and MyoPS 2024.

MyoPS 2020 [49,50] is a publicly available dataset for cardiac anatomical structures and pathological lesions, and it has been recognized by MICCAI. Due to challenges such as privacy concerns, ethical considerations, and technical difficulties, publicly available cardiac imaging datasets remain very limited. This dataset contains multi-sequence CMR images from 45 patients with acute myocardial infarction (MI), with 25 cases used for training and 20 cases used for testing. The imaging sequences include *bSSFP*, *LGE*, and *T*2. Typically, each CMR sequence contains 2 to 6 slices, with an in-plane resolution of 0.75×0.75 mm, and image sizes ranging from 412×408 to 512×515. All three multi-slice sequences were acquired during the same cardiac phase in the short-axis view of the ventricles using a breath-hold technique. The ground truth was established by averaging slice-by-slice manual delineations from three independent and well-trained observers, resulting in standard segmentation labels for scar, edema, myocardium (Myo), left ventricle (LV), and right ventricle (RV) in each image. Multi-modal images of all patients were aligned to a common space using the MvMM3 tool.

MyoPS 2024 [11,49,50,51] is provided by the MyoPS++ track of the CARE2024 challenge, including both unaligned data and versions aligned to a common space using the MvMM3 tool. The dataset consists of multi-sequence magnetic resonance scans from 250 patients collected from seven medical centers, including end-diastolic late gadolinium enhancement (*LGE*) MR images, *T*2-weighted MR images, and *bSSFP* cine MR images. However, not all sequences are available for every case. In this study, we selected 95 MS-CMR scans from Centers B and C that contain all three modalities, namely *bSSFP*, *LGE*, and *T*2. Each image is annotated with labels for scar, edema, Myo, LV, and RV. Among these, 65 cases were randomly selected for training and 30 cases for testing. To ensure reproducibility, a fixed random seed was used during the dataset partitioning process.

Data preprocessing largely determines the convergence speed and generalization ability of the model. All paired images were split into 2D slices as network inputs and normalized using z-score normalization to alleviate the problem of inconsistent intensity distributions in image data. The slices were then cropped to a uniform size of 192×192 pixels. To prevent overfitting, data augmentation strategies including random rotation and random flipping were adopted to enrich the diversity of the training images.

### 4.2. Implementation Details

Our DSS-Net was implemented in PyTorch 2.9 and trained on an NVIDIA GeForce RTX 4090 GPU with 24 GB of memory. We used the Adam optimizer to update the model parameters, with an initial learning rate of $1\times 10^{-4}$ and a weight decay of $5\times 10^{-4}$. The learning rate was adjusted using a cosine annealing strategy over 20 iterations. The model was trained for 200 epochs by randomly sampling paired images from each dataset with a batch size of 16. The loss weights were set to $\lambda_{s}=1$ and $\lambda_{se}=1$.

### 4.3. Evaluation Metrics

In our experiments, we adopted Dice score, Hausdorff distance (HD), Average Symmetric Surface Distance (ASD), Accuracy (ACC), Sensitivity (SEN), and Specificity (SPE) for comprehensive evaluation, thereby enabling an in-depth analysis of the regional and boundary similarities between the segmentation results and the ground truth. Dice score, Accuracy, Sensitivity, and Specificity are region-based metrics used to measure overlap and classification performance, whereas HD and ASD are the boundary-based metric that are particularly sensitive to segmentation outliers.

### 4.4. Baseline Methods

To validate the superiority of the proposed model, we conducted comparative experiments against a variety of representative image segmentation methods, including U-Net, U-Net++, Attention U-Net, nnU-Net, MyoPS-Net, and ACC-UNet, and further compared our method with top-ranked approaches from the MyoPS 2020 Challenge.

(1) U-Net [18]: A typical encoder-decoder segmentation network that fuses shallow detailed features and deep semantic features through skip connections, thereby effectively improving boundary recovery in medical image segmentation.
(2) U-Net++ [20]: An improved nested skip-connection segmentation network based on U-Net, which enhances multi-scale feature fusion by reducing the semantic gap between encoder and decoder features, thus improving segmentation accuracy.
(3) Attention U-Net [52]: A U-Net variant that introduces attention gates to adaptively emphasize salient features relevant to the target region while suppressing irrelevant background responses, thereby improving the localization and segmentation of key regions.
(4) nnU-Net [53]: A self-configuring medical image segmentation framework that can automatically adapt network architecture, preprocessing, training, and postprocessing strategies according to the characteristics of a given dataset, showing strong robustness and generalization ability across various medical segmentation tasks.
(5) MyoPS-Net [49]: A multimodal medical image segmentation network designed for myocardial pathology segmentation, which jointly exploits the complementary information from different cardiac magnetic resonance modalities to achieve collaborative segmentation of the myocardium and pathological regions.
(6) ACC-UNet [54]: An improved U-Net model with an enhanced convolution structure, which can enhance feature interaction and context modeling while maintaining the efficiency of CNNs, has achieved outstanding performance in medical image segmentation.

## 5. Results

### 5.1. Comparison with Existing Methods

**Figure 4:**
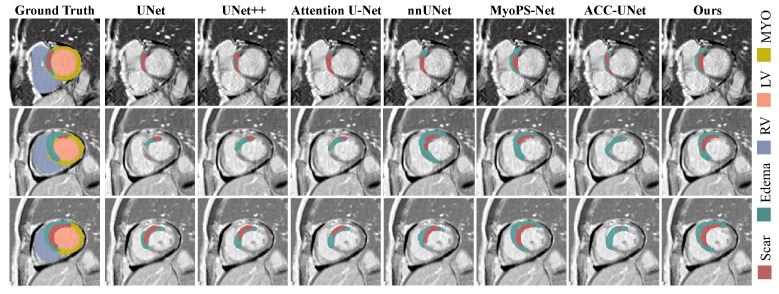
Visual comparison of myocardial pathology segmentation results of different methods on the MyoPS 2020 dataset, overlaid on *LGE* images. In the ground-truth column, five annotated tissue classes are displayed, including myocardium (MYO), left ventricular blood pool (LV), right ventricular blood pool (RV), scar, and edema. For all segmentation results, scar and edema regions are highlighted in red and green, respectively.

**Figure 5:**
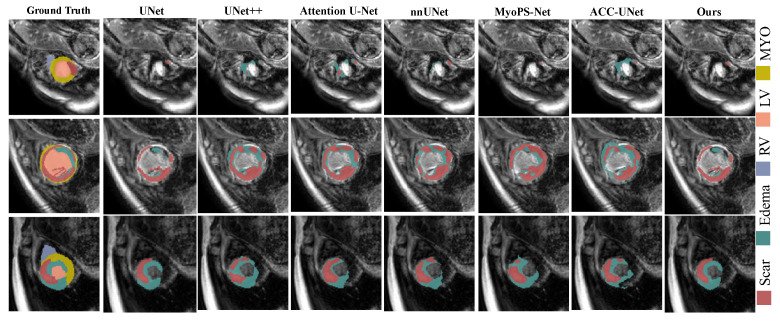
Visual comparison of myocardial pathology segmentation results of different methods on the MyoPS 2024 dataset, overlaid on *LGE* images. In the ground-truth column, five annotated tissue classes are displayed, including myocardium (MYO), left ventricular blood pool (LV), right ventricular blood pool (RV), scar, and edema. For all segmentation results, scar and edema regions are highlighted in red and green, respectively.

Table 1 and Table 2 present the quantitative results of the comparative experiments for different models, while Figure 4 and Figure 5 illustrate the visual comparison of segmentation results. Overall, on the MyoPS 2020 dataset, our method demonstrates strong competitiveness in terms of scar (0.706), edema (0.753), and the average score (0.730). Specifically, DSS-Net achieves the best Dice scores for both scar and edema segmentation, with clearer boundaries in scar regions and fewer false positives.

Although the traditional convolutional network U-Net can roughly localize pathological regions in some cases, it often suffers from missed lesions and discontinuous boundaries, especially in scar segmentation, where the predictions tend to be either fragmented or overly smoothed. This indicates that traditional single-modality or simply fused methods have clear limitations in modeling complex pathological regions. On this basis, U-Net++ and Attention U-Net improve the segmentation performance to some extent through architectural modifications. U-Net++ enhances feature reuse through dense skip connections; however, it still mainly performs progressive fusion of features at similar scales and lacks explicit modeling of cross-scale semantic differences, making it prone to blurred or adhesive boundaries in complex regions. Attention U-Net introduces attention mechanisms to strengthen responses in salient regions, but this mechanism mainly relies on single-modality or local feature distributions and therefore struggles to effectively distinguish pathological signals with large variations across modalities, resulting in false positives or missed detections in some samples.

Further comparison with the strong baseline nnU-Net shows that, although nnU-Net achieves stable performance across various medical image segmentation tasks through automated configuration and robust design, it is still insufficient in exploiting complementary information across modalities in the multi-modal myocardial pathology segmentation scenario. Similarly, although ACC-UNet enhances contextual representation and feature interaction, it remains a general-purpose convolutional model and does not explicitly model pathology-specific multi-modal cues, which limits its performance on scar and edema segmentation. In contrast, DSS-Net employs the Modality Dynamic Fusion Module (MDFM) to adaptively select pathology-relevant modal features, thereby improving the discriminative ability for scar regions more effectively. Compared with MyoPS-Net, which was specifically designed for the MyoPS task, DSS-Net improves the Dice score of scar segmentation by 4.9%, further demonstrating the advantage of the proposed method in modeling small pathological targets. This result indicates that our method significantly enhances scar segmentation performance while maintaining stable edema segmentation, thereby achieving a better balance across different pathological categories.

In addition, we compared the proposed method with the top-ranked approaches in the MyoPS 2020 Challenge, and the results are shown in Table 3. Our method achieves performance close to the leading approaches in key metrics such as Scar Dice, while maintaining a more balanced trade-off between SEN and SPE. This indicates that the proposed method attains high segmentation accuracy without sacrificing specificity for higher recall, thereby achieving a better balance across different evaluation metrics. It is worth noting that UESTC, FZU, and NJUST also adopt two-stage segmentation strategies. In comparison, our method further introduces modality dynamic fusion and stage feature aggregation mechanisms on top of this framework, enabling more effective exploitation of pathology-relevant modal information and enhanced representation of small lesions, thus achieving more stable and balanced segmentation results.

To further evaluate the computational cost introduced by the proposed two-stage framework, we compared the model complexity, training time, and inference time with several representative segmentation methods, as summarized in Table 4.

As expected, the proposed method introduces additional parameters compared with conventional single-stage networks such as UNet, UNet++, Attention UNet, and ACC-UNet, due to the incorporation of the coarse-to-fine segmentation strategy and the proposed feature enhancement modules. Specifically, our model contains 78.51 M parameters, which is larger than most baseline methods but still considerably smaller than MyoPS-Net (119.28 M). Regarding computational efficiency, although the proposed framework requires the longest inference time (30.03 ms per image), the inference latency remains within a practical range for clinical applications. Moreover, the training time of our method is only 1.92 h, which is lower than all compared methods. This can be attributed to the ROI localization mechanism in the first stage, which effectively reduces the amount of redundant background information processed during optimization and facilitates faster convergence. Overall, the proposed method achieves substantial improvements in scar and edema segmentation accuracy at the expense of a moderate increase in model complexity and inference cost.

### 5.2. Ablation Studies

#### 5.2.1. Module Abolition Analysis

**Figure 6:**
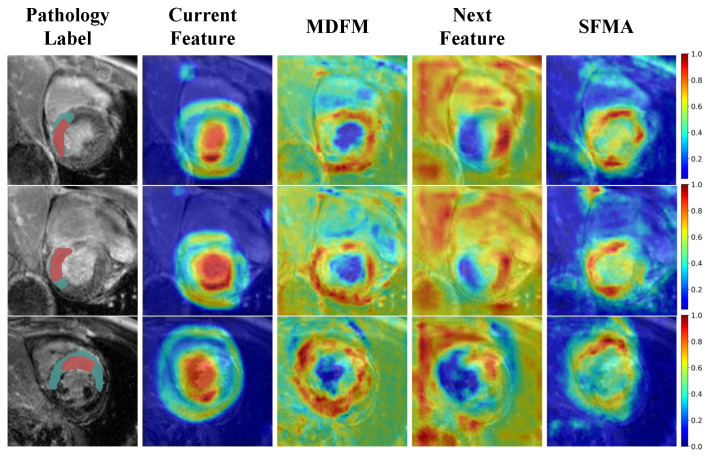
Visualization of encoder feature responses at the third stage. The first column shows the pathology labels, the second column shows the stage features before module enhancement, the third column shows the features processed by MDFM, the fourth column shows the features from the next encoding stage, and the fifth column shows the output features aggregated by SFAM. Colors from blue to red indicate feature responses from weak to strong, with more concentrated red regions indicating stronger attention to the corresponding locations.

To verify the contribution of each key component, we conducted systematic ablation experiments on the MyoPS2020 dataset, and the results are reported in Table 5. In addition, we visualized the feature responses at the third stage of the encoder, as shown in Figure 6, to further examine the effect of different modules on feature representation. When using a single-stage U-Net as the baseline, the model achieves relatively low Dice scores and large HD values, indicating limited ability to detect pathological regions and a tendency toward conservative predictions. This observation is also consistent with the visualization results: although the features can roughly focus on the left ventricle and myocardium, the pathological responses remain coarse, and some high activations are still distributed inside the cavity or around the myocardium. After introducing the two-stage segmentation framework, all evaluation metrics improve significantly. This suggests that the coarse stage provides effective anatomical priors and ROI constraints, which not only reduce redundant background interference but also guide the fine stage to learn more discriminative features within a more stable myocardial search space.

On top of the two-stage framework, MDFM and SFAM further improve the model from different perspectives. After incorporating MDFM (Row 4), the Scar Dice and SEN are further increased. As shown in the visualization, the originally scattered high-response regions become more concentrated in pathology-related areas, and the ring-like responses around lesions are more evident. This indicates that MDFM can adaptively emphasize modality features that are more relevant to pathological patterns, thereby enhancing the sensitivity to small and low-contrast lesions. However, in some cases, the activated regions still show a slight expansion tendency, which is consistent with the increase in SEN and the slight decrease in SPE, suggesting that stronger detection capability may come at the cost of mild over-response.

In contrast, SFAM (Row 5) brings more obvious improvements in boundary-related metrics such as HD, while maintaining relatively high SEN, indicating its stronger effect on lesion integrity and boundary localization. As illustrated in Figure 6, the aggregated features after SFAM exhibit a more continuous and structured response in lesion regions, with a more complete myocardial ring and clearer separation between lesions and surrounding tissues. This demonstrates that SFAM effectively combines shallow spatial details with deeper semantic information, improving both local boundary delineation and regional connectivity.

When the two-stage framework, MDFM, and SFAM are integrated together (Row 6), the model achieves the best performance across all metrics. The feature responses become more concentrated on lesion regions, while the boundaries are also the clearest. These results indicate that the two-stage framework mainly provides anatomical constraints and candidate-region focusing, MDFM enhances the selective representation of pathology-relevant modal information, and SFAM further refines spatial consistency and boundary representation. Their combination forms a progressive feature modeling process from coarse localization to target enhancement and finally to fine refinement, which jointly leads to the overall performance gain.

#### 5.2.2. Two-Stage Framework Analysis

The ablation study in Section 5.2.1 has demonstrated that the two-stage framework achieves better segmentation performance than the single-stage framework. Based on this observation, we further investigated how coarse-stage information can be best utilized in the fine stage by conducting a series of comparative experiments on the MyoPS2020 dataset. The focus of this section is not to re-evaluate the effectiveness of the two-stage architecture itself, but to compare different stage-connection strategies within the unified framework equipped with MDFM and SFAM, in order to determine how coarse-stage outputs should be transferred to the fine stage for optimal pathology segmentation.

The results are shown in Table 6. Specifically, we examined two ways of utilizing coarse-stage information: ROI cropping, which constrains the fine-stage input in the spatial domain based on the coarse segmentation result, and probability-prior guidance, which feeds the coarse-stage probability map into the fine segmentation network to provide structural guidance at the semantic level. When only ROI cropping is adopted, the model shows clear improvement over the single-stage baseline, indicating that the spatial localization provided by the coarse stage can effectively narrow the search region and reduce interference from irrelevant background. This strategy is particularly beneficial for edema, whose lesions are relatively larger and more spatially continuous. When only the probability prior is introduced, the average Dice is further improved and scar segmentation benefits more, suggesting that for small and ambiguous lesions, explicit structural priors are more effective than spatial constraints alone.

When ROI cropping and probability priors are jointly applied, the model achieves the best and most balanced performance, demonstrating that the two strategies are complementary. ROI cropping suppresses background interference by reducing the search space, whereas probability priors explicitly inject structural information to enhance pathological localization. Their combination allows the coarse-stage information to be more fully exploited, thereby improving the representation and discrimination of complex pathological regions in the fine stage. Overall, these results indicate that the connection strategy between the coarse and fine stages directly affects the final performance of the two-stage framework, and that a well-designed stage connection is critical for effectively translating coarse-stage information into fine-stage segmentation gains.

#### 5.2.3. Parameter Sensitivity Analysis of the MDFM

To analyze the effect of the compression ratio in the Modality Dynamic Fusion Module (MDFM) on module performance, we further conducted a parameter sensitivity study. According to the module design, η is used to control the channel compression ratio of the concatenated multi-modal features in the gating branch, thereby adjusting the representation dimension of the intermediate latent space. Essentially, the value of η affects the balance in MDFM between information preservation and redundancy suppression.

As shown in Table 7, when η increases from 2 to 16, the model performance on both scar and edema segmentation exhibits a steady improvement. When η is small, the intermediate channel dimension in the gating branch remains relatively high. Although this setting preserves more original information, it weakens the constraint effect of feature compression and recalibration, making it difficult to sufficiently suppress redundant responses across different modalities and thus limiting the fusion module’s ability to emphasize key pathological representations. As η increases, the intermediate representation becomes more compact, and the gating process becomes more effective at selecting highly responsive features, thereby improving the utilization efficiency of complementary multi-modal information and enhancing the discrimination of pathological regions. This effect is particularly more evident in scar segmentation, where the performance improvement is more pronounced and the fluctuation is reduced. These results indicate that a larger η enables the fusion process to focus more on discriminative responses related to pathological regions, thereby improving segmentation accuracy and model stability. Therefore, η = 16 was finally selected as the default setting for MDFM.

## 6. Discussion

Myocardial pathology segmentation is more challenging than conventional anatomical segmentation because scar and edema are usually small, irregular, and weakly contrasted, while their appearances vary substantially across imaging modalities. In this context, the proposed DSS-Net indicates that robust segmentation depends not only on stronger feature extraction, but also on whether the network can explicitly exploit anatomical constraints, selectively utilize multi-modal information, and preserve fine-grained pathological details. The overall performance of the proposed framework suggests that these three aspects are jointly important for accurate and balanced myocardial scar and edema segmentation.

A more central finding of this work is the importance of adaptive multi-modal fusion. Different CMR sequences contribute differently to scar, edema, and myocardial boundary representation, and their relevance may vary across spatial regions. This makes static fusion insufficient for fully exploiting the diagnostic value of multi-sequence CMR. By dynamically recalibrating modality-specific responses, MDFM enables the network to focus more on pathology-relevant modal cues while suppressing less informative or redundant responses. Its role is therefore not simply to combine multiple modalities, but to improve the selectivity of multi-modal representation for pathological targets.

Another key aspect is the representation of small and subtle lesions. In myocardial pathology segmentation, accurate prediction requires not only lesion detection but also preservation of structural continuity and boundary details. This is particularly difficult because repeated down-sampling may weaken local lesion responses and blur lesion boundaries. SFAM addresses this issue by strengthening the interaction between shallow spatial information and deeper semantic features, thereby improving lesion integrity and boundary delineation. Together, MDFM and SFAM form a complementary mechanism: the former enhances pathology-aware modal selection, while the latter refines the spatial organization and structural consistency of lesion features.

Overall, the proposed framework can be viewed as multi-stage and multi-scale framework that combines anatomical constraint, selective pathology-aware fusion, and fine structural refinement. This design is more consistent with the nature of myocardial pathology segmentation than directly applying a generic segmentation backbone, and it highlights that effective multi-sequence CMR analysis requires not only coarse localization, but also task-specific modeling of cross-modal information and small-lesion characteristics.

Several limitations should also be acknowledged. First, although the framework is trained in an end-to-end manner, the two-stage design contains two subnetworks, resulting in a relatively large number of parameters and high memory consumption. Second, MDFM adopts a lightweight gating mechanism consisting of two convolutional layers to achieve adaptive cross-modal feature fusion with moderate computational cost. Although this design has demonstrated effectiveness in our experiments, it may not fully capture more complex inter-modality dependencies. More advanced fusion strategies, such as deeper gating structures or attention-based mechanisms, could potentially further improve feature interaction and deserve future investigation. Moreover, the current framework assumes the availability of fully aligned multi-modal inputs. In real clinical environments, however, missing modalities, registration inaccuracies, and variations in image quality are frequently encountered. Since the proposed MDFM explicitly exploits complementary information from *bSSFP*, *LGE*, and *T*2 images, the absence of a modality may reduce the availability of pathology-specific cues. Furthermore, although MyoPS 2020 and MyoPS 2024 are representative public benchmarks, the overall sample size is still limited, and the generalization capability of the model in broader clinical scenarios remains to be further validated. Future work may therefore focus on reducing model complexity, developing more effective cross-modal fusion mechanisms, improving robustness to incomplete or imperfect multi-modal inputs, and validating the proposed method on larger multi-center datasets.

## 7. Conclusions

In this work, we propose DSS-Net, a dynamic synergistic segmentation network for myocardial pathology segmentation from multi-sequence CMR images. By combining a coarse-to-fine framework with the Modality Dynamic Fusion Module (MDFM) and the Stage Feature Aggregation Module (SFAM), the proposed method enables anatomically guided lesion localization, adaptive multi-modal feature selection, and fine-grained pathological representation. This design allows DSS-Net to effectively address several key challenges in myocardial scar and edema segmentation, including heterogeneous modal characteristics, severe class imbalance, and the difficulty of delineating small and ambiguous lesions. Experiments on the MyoPS 2020 and MyoPS 2024 datasets demonstrate that DSS-Net achieves competitive and balanced performance for both scar and edema segmentation. The comparative and ablation studies further verify the effectiveness of the proposed design, showing that adaptive modality fusion and cross-stage feature aggregation play important roles in improving lesion discrimination and structural consistency. These results emphasize the value of combining anatomical priors with pathology-aware multi-modal learning for myocardial pathology analysis.

## Figures and Tables

**Table 1:** Quantitative comparison of different segmentation methods on the MyoPS 2020 dataset in terms of average Dice score. The best performance is marked in **bold**.

Method	Scar	Edema	Avg
UNet	0.544 ± 0.268	0.600 ± 0.153	0.572
Unet++	0.587 ± 0.247	0.648 ± 0.142	0.618
Attention U-Net	0.532 ± 0.258	0.599 ± 0.165	0.566
nnUnet	0.645 ± 0.236	0.690 ± 0.128	0.668
MyoPS-Net	0.647 ± 0.258	0.722 ± 0.135	0.685
ACC-UNet	0.546 ± 0.243	0.615 ± 0.116	0.581
Ours	**0.706 ± 0.085**	**0.753 ± 0.078**	**0.730**

**Table 2:** Quantitative comparison of different segmentation methods on the MyoPS 2024 dataset in terms of average Dice score. The best performance is marked in **bold**.

Method	Scar	Edema	Avg
UNet	0.534 ± 0.273	0.593 ± 0.148	0.564
Unet++	0.576 ± 0.255	0.636 ± 0.146	0.606
Attention U-Net	0.519 ± 0.257	0.601 ± 0.160	0.560
nnUnet	0.638 ± 0.228	0.688 ± 0.132	0.663
MyoPS-Net	0.651 ± 0.246	0.724 ± 0.139	0.688
ACC-UNet	0.528 ± 0.251	0.602 ± 0.121	0.565
Ours	**0.691 ± 0.110**	**0.742 ± 0.125**	**0.717**

**Table 3:** Quantitative results of scar and edema segmentation of the top-ranked teams in the MyoPS 2020 challenge on four evaluation metrics, provided by the challenge organizers.

Team	Scar				Edema			
	Dice	ACC	SEN	SPE	Dice	ACC	SEN	SPE
UESTC [44]	0.71 ± 0.19	0.87 ± 0.08	0.74 ± 0.19	0.93 ± 0.05	0.73 ± 0.11	0.80 ± 0.10	0.72 ± 0.13	0.85 ± 0.10
UBA [48]	0.70 ± 0.19	0.85 ± 0.08	0.79 ± 0.18	0.87 ± 0.07	0.70 ± 0.13	0.76 ± 0.10	0.75 ± 0.15	0.77 ± 0.10
NPU [55]	0.68 ± 0.24	0.86 ± 0.11	0.73 ± 0.25	0.90 ± 0.10	0.71 ± 0.12	0.78 ± 0.11	0.70 ± 0.15	0.82 ± 0.13
USTB [56]	0.67 ± 0.26	0.85 ± 0.10	0.76 ± 0.26	0.87 ± 0.09	0.69 ± 0.15	0.75 ± 0.14	0.74 ± 0.16	0.74 ± 0.18
UHW [57]	0.65 ± 0.20	0.85 ± 0.09	0.70 ± 0.23	0.89 ± 0.11	0.67 ± 0.14	0.74 ± 0.10	0.72 ± 0.19	0.74 ± 0.17
FZU [46]	0.63 ± 0.22	0.85 ± 0.09	0.63 ± 0.22	0.93 ± 0.04	0.69 ± 0.12	0.78 ± 0.08	0.66 ± 0.15	0.84 ± 0.08
NJUST [58]	0.66 ± 0.24	0.88 ± 0.07	0.64 ± 0.27	0.95 ± 0.03	0.60 ± 0.20	0.77 ± 0.09	0.50 ± 0.21	0.94 ± 0.06
CQUPT [45]	0.64 ± 0.23	0.86 ± 0.08	0.63 ± 0.22	0.94 ± 0.05	0.66 ± 0.14	0.77 ± 0.10	0.61 ± 0.18	0.86 ± 0.11
Ours	0.71 ± 0.08	0.86 ± 0.07	0.73 ± 0.18	0.89 ± 0.01	0.75 ± 0.07	0.79 ± 0.06	0.78 ± 0.05	0.79 ± 0.07

**Table 4:** Computational complexity comparison of different segmentation methods.

Method	Parameters (M)	Training Time (h)	Prediction Time (ms)
UNet	17.27	4.35	3.67
Unet++	9.32	5.12	5.93
Attention UNet	15.61	2.97	5.65
nnUnet	29.97	2.37	13.73
MyoPS-Net	119.28	4.03	17.22
ACC-UNet	17.84	8.19	23.31
Ours	78.51	1.92	30.03

**Table 5:** Quantitative evaluation results of the ablation study on the proposed modules using five metrics for comprehensive assessment of segmentation performance. Two Stages: two-stage segmentation strategy. MDFM: Modality Dynamic Fusion Module. SFAM: Stage-wise Feature Aggregation Module. A checkmark (✓) indicates that the corresponding module is enabled.

Two Stages	MDFM	SFAM	Scar					Edema				
			Dice	HD	ACC	SEN	SPE	Dice	HD	ACC	SEN	SPE
			0.54 ± 0.16	18.20 ± 12.84	0.77 ± 0.11	0.60 ± 0.24	0.86 ± 0.04	0.60 ± 0.09	17.91 ± 11.64	0.72 ± 0.07	0.70 ± 0.08	0.70 ± 0.08
	✓	✓	0.58 ± 0.15	16.82 ± 12.24	0.78 ± 0.09	0.64 ± 0.22	0.87 ± 0.03	0.65 ± 0.08	16.27 ± 11.15	0.74 ± 0.06	0.73 ± 0.07	0.72 ± 0.07
✓			0.61 ± 0.14	14.55 ± 11.16	0.80 ± 0.09	0.61 ± 0.21	0.90 ± 0.02	0.70 ± 0.07	14.60 ± 10.72	0.75 ± 0.04	0.78 ± 0.04	0.74 ± 0.07
✓	✓		0.63 ± 0.12	16.36 ± 12.10	0.79 ± 0.10	0.72 ± 0.26	0.85 ± 0.03	0.72 ± 0.08	19.45 ± 13.80	0.76 ± 0.05	0.78 ± 0.08	0.77 ± 0.08
✓		✓	0.68 ± 0.07	12.57 ± 11.31	0.82 ± 0.05	0.70 ± 0.17	0.89 ± 0.03	0.71 ± 0.04	13.73 ± 11.34	0.74 ± 0.04	0.82 ± 0.05	0.72 ± 0.05
✓	✓	✓	0.71 ± 0.08	10.30 ± 10.48	0.86 ± 0.07	0.73 ± 0.18	0.89 ± 0.01	0.75 ± 0.07	16.40 ± 12.88	0.79 ± 0.06	0.78 ± 0.05	0.79 ± 0.07

**Table 6:** Results of the ablation study on stage-connection strategies in the two-stage framework, evaluated by Dice score. Based on the best-performing network, the connection manner between the coarse and fine segmentation networks was modified. Coarse segmentation: the coarse segmentation network is enabled. ROI cropping: ROI cropping is used in the fine segmentation stage. Prior probability: the probability prior from the coarse segmentation stage is used in the fine segmentation stage. A checkmark (✓) indicates that the corresponding module is enabled.

Coarse Segmentation	ROI Cropping	Prior Probability	Scar	Edema	Avg
			0.586 ± 0.151	0.655 ± 0.083	0.621
✓	✓		0.664 ± 0.112	0.702 ± 0.068	0.683
✓		✓	0.678 ± 0.097	0.695 ± 0.074	0.687
✓	✓	✓	0.706 ± 0.085	0.753 ± 0.078	0.730

**Table 7:** The influence of the MDFM module channel compression coefficient η on the segmentation performance.

η	Scar	Edema	Avg
2	0.684 ± 0.097	0.736 ± 0.084	0.71
4	0.692 ± 0.091	0.744 ± 0.081	0.718
8	0.699 ± 0.088	0.749 ± 0.079	0.724
16	0.706 ± 0.085	0.753 ± 0.078	0.730

## Data Availability

No new data were created or analyzed in this study. The datasets used in this study were obtained from third-party challenge organizers. Restrictions apply to the availability of these data. Access to the MyoPS 2020 dataset requires agreement to the user terms and submission of the signed registration form to the organizers via the official website (https://zmiclab.github.io/zxh/0/myops20/, accessed on 14 June 2026). Access to the MyoPS 2024 dataset requires registration and approval by the organizers through the official challenge website (https://www.zmic.org.cn/care_2024/track4/, accessed on 14 June 2026).
